# Introductory Lecture Delivered at the Medical Department of Niagara University, Session of 1888

**Published:** 1888-10

**Authors:** Henry D. Ingraham

**Affiliations:** Professor of Diseases of Women and Children


### *INTRODUCTORY LECTURE DELIVERED AT THE MEDICAL DEPARTMENT OF NIAGARA UNIVERSITY, SESSION OF 1888.*

By HENRY D. INGRAHAM, M. D.,

Professor of Diseases of Women and Children.

The choice of a subject for an address, such as I have, this evening, the honor of delivering to you, is generally a matter of considerable difficulty. The audience is usually somewhat mixed—that is, a part of it is professional, a portion hopes to be considered such in the future, while others have but little knowledge of medicine in any respect.

To interest you all is not an easy task, but to give you a few facts in regard to the growth and progress of the science and art of medicine, its condition in the past and present, may not be wholly unprofitable to you.

It is evident that medicine must have had a very early origin, for mankind in the most uncivilized ages would necessarily be exposed to a variety of accidents as well as disease, and would gradually learn the means of alleviating the pains, or preventing the consequences of the more external injuries, if nothing more. Yet the history of medicine is so involved in fable, and so mixed with pagan mythology, that it is impossible to arrive at the knowledge the ancients had of this or any other branch of science.

The Egyptians, however, appear to have been the first who cultivated medicine.

To the deity Isis—the wife and sister of Osiris—a peculiar medical power was attributed, and diseases were regarded as the effects of her anger. She had given proof of her power in restoring her son Orus to life. The Egyptians attributed to her the discovery of several remedies, and believed that she possessed almost, if not quite, unlimited power in medicine. Even at the time of Galen, the materia medica contained several compound remedies which bore her name.

Temples were erected in her honor, and in the morning a species of resin was burned, myrrh at noon, and in the evening a compound formed of sixteen drugs. After these preparations the sick was made to sleep in these temples, in order that the oracle might reveal to them, during sleep, the means to which recourse ought to be had for their cure.

Aside from this family, the Egyptians revered a number of other gods, and as soon as a method of making paper was discovered, the knowledge of these gods was collected and made into a book called the Embre. This book is said to have contained the rules of medical science, as taught by the most celebrated gods.

When the physicians adhered strictly to the regulations, they were held free from blame, even though the patient died; but if they wandered from them, they were punished with death, whatever might be the issue of the disease.

As diseases were considered to be the effects of the anger of the gods, they could not be cured until their wrath was appeased. The awe with which the gods were regarded by the diseased required the aid of mediators, who might implore pardon for them. Hence, the practice of medicine naturally fell to the priests, and was nothing more nor less than an absurd worship paid to the different divinities.

They concealed the medicines—which were worthless—that they administered, and medicine was esteemed a secret, the knowledge of which was vouchsafed by the gods to their favorites only.

Some of their hygienic requirements, however, are not to be despised even in this enlightened age.

Such was the condition of the art of medicine throughout the known world at that time. And it is doubtful if the Grecian Æsculapius did much to improve it, as his remedies consisted of prayers to the gods, songs, agreeable drinks, and external remedies.

It was in the temples that he and his descendants performed their most wonderful cures; and here the most strict hygienic rules then known were followed.

The hopes of the patient were raised by the recital to him of cures in cases similar to his own. If the patient had sufficient faith, he recovered. If the result was unsatisfactory, it was attributed to his want of confidence or of obedience.

So, along down through the centuries medicine was much the same in all countries. The practice was mostly, though not altogether, confined to the priests.

The Roman censor, Cato, who lived 150 B. C., was a physician, and his practice, although different in some respects from the Egyptians and Grecians, was yet but little, if any, more scientific.

Hippocrates, who lived from 460–370 B. C., is justly considered the father of medicine. He descended from a line of physicians and inherited the instructions of his father and grandfather. By carefully watching the operations of nature, he rejected the errors of former ages, freed medicine from false theories and founded for it new and solid systems.

Although many of his ideas are crude and unreasonable, yet he was the first who investigated disease and attempted to ascertain its cause or relieve its effects scientifically.

Many of his sayings are worthy of the brightest physicians of to-day.

He taught physicians that their first duty is to observe carefully the progress of nature. He demonstrated the non-utility of theories, and proved that observation alone is the basis of medicine; rules that cannot be improved even at the present day.

The course traced and pursued by Hippocrates with so much success was soon departed from by his followers. They were incompetent to profit by his instruction, and the science of medicine soon began to retrograde, and ere long was in nearly the same condition as before his time.

Considerable advancement was made, especially by the physicians and surgeons of Alexandria, before the beginning of the Christian era. About this time Greece had lost her prestige, and Rome was the mistress of the world; it was in her domains that medicine, with all the other arts and sciences, flourished to a greater extent than anywhere else. It was then that Celsus, who is called the Cicero of medicine, published his works. It is not known whether he ever practised as a physician or surgeon or not, but he has written some most excellent works, and some of his surgical precepts may be followed with advantage even now.

The practice of medicine and surgery was mostly by men who made that their business, and not by priests. It was then the custom for the physician to take his students with him when called to the bedside of his private patients. The Roman poet, Martial, said:

> "I send for Symmachus; he's here,
> A hundred pupils following in his rear;
> They feel my pulse with hands as cold as snow,
> I had no fever then; I have it now."

Of all the physicians of antiquity, the most brilliant and learned was Galen, who was born in Asia Minor in 131 B. C. Although vain and egotistical, his writings were most implicitly followed for nearly sixteen hundred years.

For several centuries the school at Alexandria was the most prominent of any, and all physicians of eminence were educated there. During a short period, however, the Nestorians maintained a medical school at Odessa under the protection of the Persian Empire, but this school was comparatively short-lived.

The Arabians made considerable advancement in chemistry and pharmacy from the sixth to the twelfth century; and it is to this people that we are indebted for our earlier knowledge of the subject. But the practice of the Arabian physicians and surgeons was most gross and superstitious.

After the sixth century, the practice of medicine and surgery in Europe was confined almost exclusively to the monks as a work of piety and charity, and as a duty attached to their divine calling. Having but little scientific knowledge, and never investigating causes of disease or even using medicine to any extent, they were really only pious and faithful nurses.

Nuns likewise engaged in the practice of medicine as one of their duties; the most celebrated and learned of the number being Hildegarde, Abbess of the convent of Rupertsburg, near Bingen. She died in 1180, and was placed with the saints for her miraculous cures of the sick.

Owing to the contempt in which the greater portion of the physicians were held, various councils of the church expressly forbade the members of the superior clergy to practice medicine. The lower clergy were allowed to continue practicing medicine, but not to perform any surgical operation.

The Benedictine monks in the kingdom of Naples did more than any others to advance medical science by the establishment of two schools, which became somewhat celebrated.

In France, during the reign of Louis IX., medicine and surgery made rapid advancement. And at Bologna, in 1315, Mondini De Luzzi, professor of anatomy, dissected the human subject before his pupils for the first time, it is asserted, in 1700 years. He subsequently published a work on anatomy, which, although imperfect, was considered classical for more than two centuries.

It was not, however, until the beginning of the sixteenth century that the practice of medicine was brought up to the standard of Hippocrates. About this time his works were translated into Latin, and they, together with those of Galen, were the principal text-books of the schools.

In 1619, Wm. Harvey discovered the circulation of blood; and since that time so many men of genius have made their appearance, and so many discoveries and improvements have been made, that the time is too brief even to mention them.

In America, during its colonial days, the practice of medicine was exercised by the clergy in connection with their religious duties. And even men high in office, as governors of provinces, devoted attention to the practice. One branch—that of obstetrics—however, being exclusively in the hands of women.

The last hundred years are remarkable for the immense amount of labor and research which has been brought to bear upon every department of medical science. Chemistry has become a subject of more scientific study. Physiology and pathology are no longer sealed books.

The microscope has revealed wonders that were never dreamed of. The discovery of ether and other anæsthetics enables the surgeon to perform many operations that otherwise could not be successful. Auscultation enables us to ascertain the condition of the heart and lungs almost as well as though we could see them. Advancement has been so great that it seems almost incredible. As a point of history, full of valuable deductions, it is thought good to look back upon the condition of medicine in former times, and to find that it has always kept pace with the progress of physical and moral sciences. Where these have been marked by folly and credulity, it has exhibited the same imperfections.

We find the men who appear to us so defective in their powers of observation and reflection, were but examples of the general bearing of the age in which they lived.

In ancient times, as with rude and uneducated nations of the present day, the physician acted as the magician. Physic was an art which was supposed to be most mysterious, and its practisers were presumed to hold communion with the world of spirits.

Many of the superstitions connected with the earlier history of medicine prevail now as they did formerly. The condition of experimental science, or rather the want of it at that time, occasioned their prevalence in the highest intellects, whilst at the present day they are mainly restricted to the ignorant and uneducated.

The belief that some human beings can attain the power of conferring good or inflicting ills on their fellow-creatures, and of controlling the operations of nature, is one of the highest antiquity. It has appeared in every region of the globe, and from its extensive prevalence, it is evident that the human mind, especially in a state of ignorance, is a soil well adapted for its reception and cultivation. Life has so many evils which the uninformed mind can neither prevent nor avert, and encourages so many hopes which every age and condition are anxious to realize, that we can hardly be astonished to find that a portion of mankind becomes the willing prey of impostors, who practice on their credulity by promise of greater good than the usual course of nature can dispense.

But mankind is wiser now than formerly, and if you think you can succeed in duping your patients, can relieve them of their ills as those who practiced medicine of old did, that it is not necessary for you to master the fundamental principles of the science of medicine, then this is no place for you. I take it for granted, however, that there are no such here. The day for those who succeed without knowledge is rapidly passing away. It is true, too true, alas! that some physicians have but little knowledge. But the number of such physicians is becoming much less every year.

You cannot succeed in practice with the same amount of knowledge to begin with that even your immediate predecessors had. There is more to learn now—a broader field to cover—a greater amount of knowledge is necessary—and a longer period of time is required to obtain it.

Medicine, as we now see it, is almost a new science. It hardly had an existence a little more than one hundred years ago. It was born with Harvey, but it slumbered until awakened by Morgagni, Auenbrugger, Jenner, Laennec, Bright, Lister, McDowell, Sims, and many others that might be mentioned.

The methods in medicine are now based on the knowledge of the fundamental branches, and not upon fancy or theory. Anatomy, physiology, chemistry, pathology, hygiene, and the improved means of observation and experiment, are the foundations upon which the science rests. And even in these branches our knowledge is progressive.

Doubtless most of you think anatomy a fixed fact—that there can be no change in this branch of medical science—that the structure of the human frame does not vary; and it is true that it does not. Yet our knowledge of anatomy is progressive, and scientific medicine may expect much from it in the future. With a few exceptions, the limitation of gross anatomy was reached some time ago. But there is yet a brilliant future for minute anatomy. We want to know more of regional medical anatomy—the anatomy of internal organs in their relations to the parts around them, which are likely to become implicated in disease.

We also need more accurate information as to nerve termination and anastomosis, both in viscera and on the skin; as to the parts of brain and spinal cord.

The attention now given to the application of electricity calls for this. Although the great bulk of the anatomical work is done, yet it is still too indefinite to serve the purpose of the clinician.

Physiology teaches us how secretion, nutrition and growth are brought about. The great problem of the functions of special centers in the brain and spinal cord is being settled, and with this advance we have gained not only in the recognition but in the cure of disease.

As one instance of the correct appreciation of symptoms, I would mention the successful removal of brain tumors, made possible only by recent physiological researches.

Doubtless, in the near future more brilliant results than are now achieved will follow the investigations in this branch of medical science.

Chemistry is continually adding new and valuable remedies to our armamentarium, and it is extracting from well-tried agents the active principles, thereby enabling us to administer medicines in much more palatable form and smaller doses.

It has taught us so much of the character of the secretions, that every intellectual physician uses the test-tube at the bed-side; and the means for its use are becoming more and more simplified each year. Perhaps one of the most wonderful as well as beneficial discoveries of chemistry is that of the poisons developed by decomposition.

It is not only that these ptomaines, which have been found in stale eggs and cheese, as well as in diseased and putrid meat and fish, explain the poisonous symptoms which we know these articles occasion, but decomposition occurs also in the human body, due either to cadaveric changes, or formed previous to death.

The system, through their presence, may poison itself, and it is likely that by their study we shall find a solution more certain than we now have of all those numerous diseases which we vaguely call blood-poisoning.

Much of the advance of medicine in late years has been due to the study of general pathology and pathological anatomy. Neither of these branches receives—at least in this country—the attention to which it is entitled; yet a knowledge of them is becoming every year more and more essential, and more and more general.

The whole medical world, dissatisfied with its knowledge of the causes of disease, particularly communicable disease, is seeking the cause; and it is believed to be due to little bodies of specific power—the so-called bacteria.

No doubt many diseases are due to this cause; though just how many, has not been fully demonstrated as yet. But science feels that if it can be demonstrated that they are the causative agents, that there will be hope of overthrowing disease in its very beginning.

It must be confessed that, thus far, as regards internal medicine, but little progress has been made in discovering agents which destroy the germs. There are no medicines which can be used to destroy them as the surgeon does with his antiseptic lotions. Yet, no doubt, the time will come when means for their destruction will be found. When has any discovery stood still? Look at all of the wonderful achievements of the past, and then say, if you can, that there will be no more progress. We are barely at the threshold of improvements and progress.

The future of medicine will be much more brilliant than anything that is now known in regard to it.

The medicine of the future will be preventive in character to a much greater extent than it is at present. The physician in the times near at hand will be a decided sanitarian. And, strange as it may seem, he will do all in his power to instruct people so to live that there will be much less need of his professional services.

Let people say what they may of the medical man—there is no other profession or class that strives as hard as he to do away with the necessity for himself.

Every physician is sensible of the growing importance of hygiene. We know the value of sunshine, of fresh air, of open spaces, of pure water, of wholesome food, of appropriate dress, of cleanliness, of effective drainage, of thorough disinfection.

We feel the necessity of saving young lives from premature exhaustion by the drain on their unformed powers, from late hours, or from overwork, especially from the senseless cramming of useless knowledge into tired frames.

We heed the exercise that soothes and refreshes and that develops the body as well as the mind.

Upon us rests the responsibility of making people generally understand all this.

But I assure you that it is no slight task. We meet ignorance, cupidity and indolence on every hand.

Do you think that there is but little to do in this direction?

In the United States alone the census returns inform us that every year not less than 200,000 deaths, or considerably more than one-fourth of the whole number, are due to epidemic diseases.

A careful estimate indicates that if proper means were taken to stamp out epidemics, that at least 100,000 of this sacrificed host could be saved.

Much of the improvement in medicine, especially in therapeutics, is due to the knowledge that many of the diseases are self-limited, and to the observation of the fact that they run their course uninfluenced by medical means.

This study of the history of disease has been of great value, and, giving a standard, it enables us to correct the claimed importance of special agencies, and to estimate at their true value the vaunted cures of exclusive systems. It is through this that we begin to comprehend the power of Nature. And yet, to insist at the present day upon the unaided power of Nature, is to be as much behind the times as to continue in the constant use of disturbing remedies.

Medicine has advanced, and it has given remedies for relief and cure, the values of which have been proven.

And those who seek counsel will turn to him who knows the limits, and who believes in the resources of his art, and uses that art boldly; and not to him who sits down simply to see what nature will do, and tries to throw the cloak of a philosopher over the shortcomings of an ignoramus and sluggard.

The practice of medicine will, doubtless, in the future be more simple, accurate and decided. Yet it will require, on the part of all who engage in the work, more labor, more study, to attain this simplicity, this accuracy, this decision.

With so much broader field before you than your predecessors had, you can readily see the necessity of a more thorough preparatory course than they had. Unless you lay the foundation broad and deep, unless you comprehend the fundamental branches, you will most likely be sad failures in your profession, or, at least, fail to attain that prominence to which your abilities justly entitle you.

This necessarily leads me to say a few words in regard to the standard of medical colleges. Many have no preliminary requirements; the student is not obliged to pass any examination before admission. Doubtless, it is hoped that he can read common English passably well. He is obliged to attend only two courses of lectures, of about five months each. He is also to furnish the certificate of some physician that he is of good moral character, and that he has studied medicine three years.

It is, however, often the case that the candidate begins the study of medicine at the beginning of one term, and graduates with the highest honors at the close of the next succeeding term. Certainly this is no fault of the student, but what can be said of a school that places its standard so low?

The laws governing medical education were made some time ago, when there was not so much to medicine as there is at present; when the student could master the principles of medicine in a much briefer period of time than now. Yet it is very doubtful if the student had much of an idea even of what little was taught in this brief period of time.

Although the science of medicine is much more comprehensive than formerly, yet the length of time required to complete a course in medicine was not lengthened in any of the colleges until within a comparatively recent date. In fact, about the beginning of the present century the requirements necessary to become a physician were greater than they are in most of the medical colleges at the present day.

Ten or fifteen years later the standard of medical education was considerably lowered, and in a short time it became so ridiculously low, that in 1847 the American Medical Association was formed, and one of its objects, as set forth in its preamble, "is to elevate the standard of medical education."

Yet the only medical college to respond to the solicitations of the American Medical Association was the oldest one in the country—the University of Pennsylvania—which extended its session of studies to six months. Not another medical college followed her example, and after six years of struggling with diminished classes, she abolished her advanced position and returned to her former requirements. So you see that about thirty years ago the attendance upon two courses of lectures, of four months each, was all that was necessary to obtain the degree of M. D. Since that time there has been a great improvement in many of the medical colleges. It is true that there has been but little advance made in some of the schools, yet several of them, and those of the best reputation, have increased their term of study, instituted examinations for admission, and adopted the graded course.

In every other country but ours, without, so far as I know, a single exception, where a system of medical education can be said to exist, certain general principles are found embodied in that system—the most important ones being these:

In the first place, the applicant, before being allowed to matriculate as a student of medicine, must pass a preliminary or entrance examination, unless he have a degree from some literary college or similar institution, or have a certificate that he has a fair knowledge of the grammar of his native tongue, of arithmetic, algebra and Latin, and, in most cases, of the elements of physics or natural philosophy.

In the second place, the student is required to devote about nine months each year for four, five, six, or even seven years, to his technical education before he is eligible for examination for the degree in medicine, the studies during this time being carefully graded. The instruction is not only didactic and theoretical in character, but the student is required to do practical work in the laboratories of anatomy, chemistry and pharmacy, while his personal training in medicine and surgery is acquired at the bedside in the hospital wards.

In the third place, the student is required to pass partial examination, at certain intervals, to determine his fitness to pass from one class to the next; and, at the close of his studies, his examination for the degree is conducted with strict impartiality by men who have no interest either in rejecting or admitting him to practice.

With the two exceptions that the term is six instead of nine months, and the course three years instead of more, I am proud to say that the above are the requirements of Niagara University. This institution, together with a few others in this country, stand as bright beacon-lights in the cause of higher medical education. A few pioneers of this kind have done much to advance medical education in our own beloved land; and, consequently, much for the benefit of its people. Whatever tends to elevate a profession, is an advantage not only to that profession but to all classes.

It may seem to you, young men who are about to engage in the study of medicine, that our requirements are too severe; that you will not be able to pass the ordeal before you. I can safely assure you that if you have fair ability, and I concede you have or you would not have the necessary education to admit you into this college, and are willing to apply yourselves with reasonable diligence during the next three school years, that you will not only pass the faculty and the board of examiners, but you will have what many young men do not have when they graduate —a good knowledge of medicine.

Gentlemen, it is necessary for you to have this knowledge to enable you to pass your examinations, and you will as certainly have it as you study.

A graded course will enable you to accomplish much more than the old method of compelling the beginner to listen to the same lecture as the student who is about to graduate.

In no educational institutions, other than medical colleges, are all classes and grades grouped together, and in no others would such a foolish and unreasonable arrangement be tolerated.

No defense of this system can be made, except that it is the quickest way to get a man's money by giving him the least possible instruction. This system is a relic of the past, and is unworthy the sanction of any respectable school.

It is certain that for a few years the University of Niagara will not have as large classes as she would under a less elevated and exacting system. Yet she is thoroughly educating her graduates, and thereby conferring a favor not only upon them but also upon the community in which they may live.

It is felt, therefore, that Niagara University, confident of success, may look forward to a most brilliant future for her Medical Department. We would appeal to all—not only her alumni, but to all who are interested in the advancement of the medical profession—to aid us by their active sympathy and co-operation. And with that support we are confident that our efforts will produce results that will be a credit to the University and an honor to her students.

